# Death of Dr. Bostock and Dr. John Thompson

**Published:** 1847-04

**Authors:** 


*Death of Dr. Bostock.*—This distinguished physician died in London in August last, at the age of 73. His disease is reported to have been *cholera*—of course of the sporadic variety. *New York Annalist.*

Dr. John Thompson, late Professor of General Pathology in the University of Edinburgh, died on the 11th of October last, in the 82d year of his age. He was eminent both as a teacher and practitioner, and is well known as an author of various works on medical science, of which his lectures on inflammation, his edition of Fourcroy, his account of the varioloid, and his life of Cullen are the most celebrated. His life was a busy and useful one. Clinical lecturing owes to him its existence in Edinburgh. He gave there the first separate course of lectures on surgery. He was one of the celebrated eight who commenced the Edinburgh Review. It was he who succeeded in inducing the College of Surgeons in Edinburgh, to found a professorship of surgery, and he delivered the course for sixteen years. He was also the founder of its museum. He established a dispensary, from which the poor were visited at their own houses. He was a warm champion of the non-mercurial treatment of Syphilis. He was President of the College of Physicians. He delivered the first lectures in Edinburgh on Pathalogical Anatomy, in preference to a symptomatology, and collected the finest collection of colored pathological drawings, with the histories of the diseases attached, now in existence, and worth $10,000. Of such a man we may safely say, that he has raised a monument more durable than brass. He was sceptical as to the power of medicines, and too honest to conceal his want of faith, which somewhat diminished his success as a practitioner. His two sons are Professors.—*Ibid.*

